# Weight gain in children from birth to 10 years on antiretroviral treatment

**DOI:** 10.4102/sajhivmed.v23i1.1413

**Published:** 2022-10-28

**Authors:** Janine Scholtz, Susanna M. Ellis, Herculina S. Kruger

**Affiliations:** 1 Centre of Excellence for Nutrition, Faculty of Health Sciences, North-West University, Potchefstroom, South Africa; 2 Department of Pure and Applied Analytics, Faculty of Natural and Agricultural Sciences, North-West University, Potchefstroom, South Africa; 3 Medical Research Council Unit for Hypertension and Cardiovascular Disease, Faculty of Health Sciences, North-West University, Potchefstroom, South Africa

**Keywords:** HIV, infants, children, weight, height, ART, growth monitoring, nutritional assessment, WAZ, catch-up growth

## Abstract

**Background:**

Inadequate weight gain could indicate clinical deterioration in infants and children living with HIV (CLHIV). The World Health Organization’s (WHO) weight-for-age z-score (WAZ) growth standards and reference charts are currently used in South Africa to assess weight gain in CLHIV on antiretroviral treatment (ART).

**Objectives:**

To assess weight gain patterns of infants and children initiated on ART and to compare weight gain patterns between the WHO WAZ growth standards and population-specific curves constructed from data of CLHIV on ART.

**Method:**

A quantitative, retrospective and descriptive-comparative design was used. The weight gain patterns of 98 infants and children from birth to 10 years old during the 24-month period following ART initiation were recorded and assessed using two different growth charts.

**Results:**

The children’s rate of weight and length gain improved significantly over 24 months since ART initiation, but complete catch-up growth was never achieved. Most (69%) of the children had increased weight gain according to the WAZ growth standard and reference charts versus only 16% according to the HIV-specific weight gain curves.

**Conclusion:**

Antiretroviral treatment improved weight and height gain in CLHIV, but the interpretations of weight gain differed significantly between the WHO chart and HIV-specific weight gain curves. Population- and treatment-specific references could improve weight monitoring in CLHIV and assist in the timeous identification of malnutrition.

## Background

HIV infection remains a global epidemic, with 37.7 million people living with HIV in 2020. An average of 1.7 million children younger than 15 years are living with HIV. An estimated 150 000 children were newly diagnosed in 2020.^1^ The provision of combination antiretroviral treatment (ART) to pregnant and breastfeeding women resulted in a 54% reduction of new infections in children.^2^ The improved coverage of ART among pregnant women in the public sector resulted in 90% of women living with HIV currently on ART.^3^ The increase in ART provision has also resulted in an increasing population of HIV-exposed uninfected infants and children.^2^

It has been established that growth reconstitution occurs following ART initiation.^4^ Research indicates that growth in HIV-exposed uninfected infants and children differs and is still lagging behind when compared to that of HIV-unexposed uninfected children.^5^ This finding could be related to an increased immune activation,^6^ changes to the gut microbiome, as well as systemic inflammation following prolonged in-utero or postnatal HIV and ART exposure, despite a negative HIV status.^7,8^ Children living with HIV (CLHIV) on ART from resource-limited settings have growth failure.^4^ Most children on ART are from resource-limited settings where malnutrition still prevails.^9,10^ In addition, several studies have shown a high prevalence of undernutrition in CLHIV, especially in sub-Saharan Africa.^11,12,13,14,15^

Malnutrition is multifactorial and impacts negatively on the growth, development and immunity of a child.^16^ In addition, malnutrition is associated with a high risk of morbidity and mortality.^17^ Furthermore, malnutrition was described as the most important risk factor for the global burden of disease.^16^ Early initiation of ART can reduce the risk of malnutrition in CLHIV, yet specific interventions are valuable to improve the nutritional care of CLHIV, especially because child malnutrition and paediatric HIV infection are two major interacting issues in sub-Saharan Africa.^18^ Anthropometrical indicators, such as weight and height or length, are common measurements that are obtained in order to assess nutritional status in children. Mid-upper arm circumference (MUAC) measurements are also assessed; however, MUAC relies on a single absolute cut-off value that is taken, irrespective of age, sex, weight or height to help identify children with existing acute malnutrition.^19^ Mid-upper arm circumference measurements may not be accurate in depicting nutritional status due to genetic variations and should rather be used in conjunction with other variables, such as weight-for-age z-score (WAZ), in guiding admission for treatment of malnourished children.^19^ Growth is strongly associated with ART treatment outcomes^20^ and it remains crucial to monitor children’s growth in order to establish their risk of malnutrition.^21^

In 2006, the World Health Organization (WHO) released growth standard charts for infants 0–60 months of age^22^ and growth reference charts for older children in 2007.^23^ The WHO standards and references are used to assess the progress of achieving the Millennium Development Goals.^24^ However, it has been argued that local growth charts might be more suitable for individual growth monitoring.^25^ It should be considered that the growth environment of CLHIV is different to that of a healthy child, due to HIV and ART exposure.

In 2015, Yotebieng et al.^20^ constructed weight gain percentile curves for CLHIV who were initiated on ART, in order to investigate the value of these curves in predicting treatment outcomes. In this study, we aim to apply these HIV-specific weight gain curves to investigate how these weight gain patterns compare to the weight gain percentile curves of the WHO WAZ growth standards and references. Furthermore, this study describes the weight changes of the study participants following their initiation of ART at their 6-month, 12-month, 18-month and 24-month follow-up visits at an ART clinic in Gauteng, South Africa.

## Research methods and design

### Study design

This study was a quantitative, descriptive-comparative study with a retrospective design. Data collection of clinic visits ranged from 2004–2007 to 2010–2015. Patient records were accessed and data pertaining to weight and length or height changes of the boys and girls that were treated with ART were collected. Mid-upper arm circumferences were not recorded in the patient files. Other parameters, such as the ART regimen, routine blood biochemistry and clinical signs that were recorded in patient files, were also collected. The children’s ART regimens at the time of treatment (between 2004 and 2015) included abacavir (ABC), lamivudine (3TC), zidovudine (AZT), lopinavir/ritonavir (LPV-r), efivarenz (EFV), stavudine (D4T) and nevirapine (NVP). Unfortunately, viral load data were incomplete in most files and were therefore not recorded; however, CD4 percentages (CD4%) could be obtained from 74% of the participant files.

### Setting

The study was conducted in 2017 at Dr George Mukhari Academic and Public Hospital ART clinic in Gauteng, South Africa. Data were collected of patient visits from 2004 up to 2015. All measurements recorded in the patient files were performed using standard operating procedures and definitions based on South African and WHO guidelines.^26,27^

### Study population and sampling

Boys and girls aged between birth and 10 years old who were initiated on ART at any time during the lifespan of this ART clinic and who attended follow-up visits for a minimum of six months and a maximum of 24 months after ART initiation, were eligible for inclusion in this study. Infants and children who received tuberculosis (TB) treatment for more than 6 months (one-quarter of the follow-up time) during the first 24 months after ART was initiated were excluded. The ART clinic’s physical filing system contained patient records of infants and children (*n* ~ 2000) who were initiated on ART and aged younger than 15 years old. The records included those who are still treated at this clinic, as well as those who have been lost to follow-up. Due to random filing, a convenience sampling method was used to locate files of eligible children until the sample size of approximately 100 files was reached.

### Data collection

Data collected of clinic visits ranged from 2004–2007 (*n* = 9) to 2008–2010 (*n* = 52) and 2010–2015 (*n* = 37). Data were captured electronically into Microsoft Excel. Birth dates were captured in order to calculate initiation and follow-up ages, as well as WAZ and length or height-for-age z-scores (L/HAZ). Demographic information, including sex, age, date of birth and ART regimens, was recorded for every follow-up visit. Progress notes, clinical or immunological staging, biochemical data, CD4% and haemoglobin (Hb) levels were also collected from the patient files.

Weight gain was the primary outcome variable in this study. In addition, the weight gain patterns of the study participants were interpreted and compared between the WHO growth standard and reference WAZ charts^22,23^ and the HIV-specific weight gain curves.^20^ Length or height measurements were also captured if recorded in the patient files; however, length or height measurements were not performed routinely.

Baseline weight at ART initiation and at least one follow-up visit that fell on a date that was either on the 6th, 12th, 18th or 24th month post ART initiation were analysed. Weight records that deviated by half a month before or after these follow-up dates were also recorded. The ART initiation age was calculated from the date of birth and date of relevant clinic visit. Microsoft Excel was used to calculate age at follow-up and weight gain for each follow-up. Weight gain was plotted onto the HIV-specific weight gain charts.^20^ These curves are specified for weight gain after ART initiation, then at 6-month, 12-month, 18-month and 24-month follow-ups.^20^ Weight gain was manually plotted onto the *y*-axis against current age (in months) on the *x*-axis. Afterwards, the percentile or percentile range for that specific plotting point was captured. Percentiles ranged from the 5th, 10th, 25th, 33rd, 50th, 75th, 90th to the 97th percentile. Percentiles and percentile ranges were coded in the following manner: (1) < 3 percentile, (2) 3rd to 9th percentile, (3) 10th to 24th percentile, (4) 25th to 32rd percentile, (5) 33rd to 49th percentile, (6) 50th to 74th percentile, (7) 75th to 89th percentile, (8) 90th to 97th percentile (9) and > 97th percentile. After coding, the growth pattern for each individual child was assessed. The growth patterns were then categorised in the following manner: (1) decreased (consistent decrease from high to lower percentiles), (2) maintained (at least three out of four weight gains were in the same percentile range), (3) increased (consistent increase from low to higher percentiles) and (4) fluctuated (no consistent pattern of increase, decrease or maintenance of weight gain).

The scales used at the ART clinic were calibrated to confirm validity of the recorded weights. The differences between displayed weight and the calibration weight ranged between 0.0 kg and 0.2 kg, indicating a 98% accuracy of the weight measurements. Recorded data were checked for outliers and were routinely corrected during duplicate data collection by confirming values of every fifth file according to the original data in the files.

### Data analysis

Descriptive statistics were used to describe the baseline and follow-up characteristics of the boys and girls. The distribution of all continuous variables was tested using histograms, Q-Q plots and the Shapiro-Wilk test. The variables of the study participants are presented as means and standard deviations (for normally distributed data) or medians and inter-quartile range (for data not normally distributed). Categorical data (gender, ART regimen, WHO stage) are presented as frequencies. Statistical significance was deemed as a *P*-value of < 0.05. Mixed linear regression was then used to test for significance of increases in weight, WAZ, Hb and CD4% across the follow-up period. Mixed methods analysis of longitudinal data was performed using the restricted maximum likelihood (REML) function with an unstructured covariance type. The quality of model fit was estimated by Akaike’s information criterion (AIC). Repeated comparisons were done to test for changes between 6-monthly follow-up visits, with Sidak adjustment for multiple comparisons. A kappa test for agreement between the four identified growth pattern categories according to the two growth norms was performed. The kappa test was also repeated in a subgroup analysis, where only children with a low WAZ at ART initiation was included, defined as WAZ < −1. All statistical analyses were performed using the Statistical Package for the Social Sciences (SPSS), version 23 (IBM Company, Armonk, New York, United States).

### Ethical considerations

Ethical approval was obtained by the North-West University Research Ethics Regulatory Committee (NWU-IRERC) (NWU ethics number: NWU-00080-16-A1).

All the data were collected from patient records only. No informed consent was required from the parents of the children. The data were captured anonymously on a password-protected device. Permission to conduct research at the ARV clinic was approved by the Chief Executive Official of hospital, and ethical clearance from the Sefako Makgatho Health Sciences University Ethics Committee (SMUREC), prior to HREC approval. This project was conducted under controlled ethical conditions at all times in accordance with the World Medical Association Declaration of Helsinki.

## Results

The data screening for inclusion eligibility is indicated in Table 1. The total number of baseline and follow-up data points included in the statistical analyses was 363, which was derived from 98 infants and children.

**TABLE 1:** Data screening, eligible data points and participants.

Inclusions	Participants	Number of data points
**Follow-up data**		
Baseline and 1 follow-up visit	12	24
Baseline and 2 follow-up visits	27	81
Baseline and 3 follow-up visits	37	148
Baseline and 4 follow-up visits	22	110

Total	98	363

**FIGURE 1:**
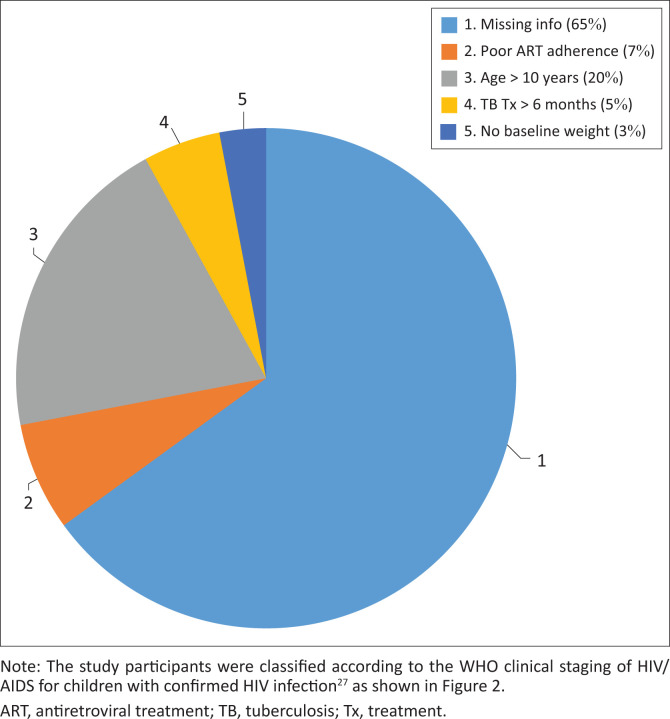
Percentage of participants that were excluded.

Figure 1 illustrates the number of children that had to be excluded. Most exclusions were made due to missing information (65%) from participants who were transferred out of this clinic, as well as participants whose follow-up visits fell too far outside of the specified follow-up periods of 6, 12, 18 and 24 months. A total of 94 files were excluded from this study, while 98 files were included for analyses.

The ART regimen of 14% of the infants and children was changed at some point during the 24-month follow-up period. Most children were treated with the following ART combinations: ABC, 3TC and LPV/r (46%), or 3TC, EFV and D4T (40%). Data collected of clinic visits ranged from 2004–2007 to 2010–2015, therefore the ART regimens varied based on the first-line and second-line regimens followed at the time. At baseline 2004–2007 most children ≥ 3 years (and ≥ 10 kg) were on 3TC, D4T and EFV, while younger children were on 3TC, D4T and LPV-r. With time children under 3 years (or < 10 kg) were switched to ABC + 3TC and LPV-r, while older children were switched to ABC, 3TC and EFV. Small numbers of children were on ABC, 3TC and NVP, D4T, 3TC and EFV/NVP, AZT, 3TC and LPV-r/NVP, or AZT, ABC and LPV-r.

Table 2 displays baseline and follow-up characteristics of the boys and girls. The girls were significantly older than the boys at ART initiation. Weight increased significantly from ART initiation to 24 months, and also at each of the 6-month intervals compared to baseline (*P* < 0.0001; AIC = 1484.9). Length or height-for-age z-scores did not increase significantly from ART initiation to 6-months follow-up (*P* = 0.058), but increased significantly from baseline to 12-month, 18-month and 24-month visits (*P* ≤ 0.001; AIC = 895.3). The low mean baseline WAZ (−2.1) falls into the underweight-for-age range. The largest mean improvement was at the 18-month follow-up point with a change from WAZ of −2.1 to 0.9. Weight-for-age z-score increased significantly from ART initiation to 24 months, and also at each follow-up visit compared to baseline (*P* < 0.0001; AIC = 970.3).

**TABLE 2:** Characteristics of the study participants.

Participants	All children			Boys			Girls			*P*
	*N*	Mean	s.d.	*N*	Mean	s.d.	*N*	Mean	s.d.	
Initiation age (months)	98	35.5	27.6	54	30.0	24.9	44	42.4	29.3	0.03*
Initiation weight (kg)	98	10.8	4.5	54	10.3	4.2	44	11.5	4.8	0.18
Initiation WAZ	97	−2.1	1.5	48	−2.0	1.6	31	−2.3	1.6	0.37
WAZ at 6 months FU	58	−1.3	1.1	24	−1.4	1.1	20	−1.0	1.4	0.31
WAZ at 12 months FU	58	−1.0	1.0	29	−1.0	1.0	16	−0.9	1.3	0.90
WAZ at 18 months FU	79	−1.0	0.8	35	−0.9	0.9	18	−1.0	1.0	0.78
WAZ at 24 months FU	70	−1.1	0.9	25	−1.1	0.9	17	−1.0	1.2	0.74
L/HAZ at initiation	92	−2.3	1.2	51	−2.3	1.3	41	−2.2	1.2	0.71
CD4+ (%)	73	15.0	8.8	37	14.7	8.9	36	15.8	8.7	0.58
Hb (g/dL)	68	9.7	1.4	38	9.7	1.4	30	9.7	1.4	0.84

Note: WAZ and L/HAZ classified according to World Health Organization growth standards.

*n*, number of participants; s.d., standard deviation; FU, follow-up; WAZ, weight-for-age z-score; L/HAZ, length or height-for-age z-score; Hb, haemoglobin.

* , Statistically significant difference between boys and girls.

Both haemoglobin and CD4% increased significantly from ART initiation to 24 months, including between each follow-up visit compared to baseline (AIC = 723.1 and AIC = 1655.3; *P* < 0.0001). There were no significant differences in prevalence of being underweight (*P* = 0.43) or stunting (*P* = 0.68) at initiation of ART between the boys and girls. The mean L/HAZ score at initiation was −2.3, with 55.1% of the participants being stunted when ART was started.

Figure 3 depicts the agreement between weight gain pattern categories according to two different references: HIV-specific weight gain curves^20^ versus the WHO WAZ growth standard and reference charts.^22,23^ The number of participants (*n* = 86) includes those who had at least two follow-up visits after ART initiation. According to the HIV-specific weight gain curves, most (62.8%) of the participants remained within the same percentile range of weight gain (maintain), compared to only 19.8% according to the WHO reference. The weight gain of 16.3% of the participants decreased across the percentiles according to the HIV-specific weight gain curves, while only 10.5% of the participants’ weight gain decreased from a higher to a lower percentile according to the WHO reference. The same proportion of participants showed an increase in their weight gain (16.3%) according to the HIV-specific weight gain curves, while according to the WHO reference most of the participants (68.9%) showed weight gain. The proportion of participants with fluctuating weight gain patterns was low according to both references (4.6% and 1.2%).

**FIGURE 2:**
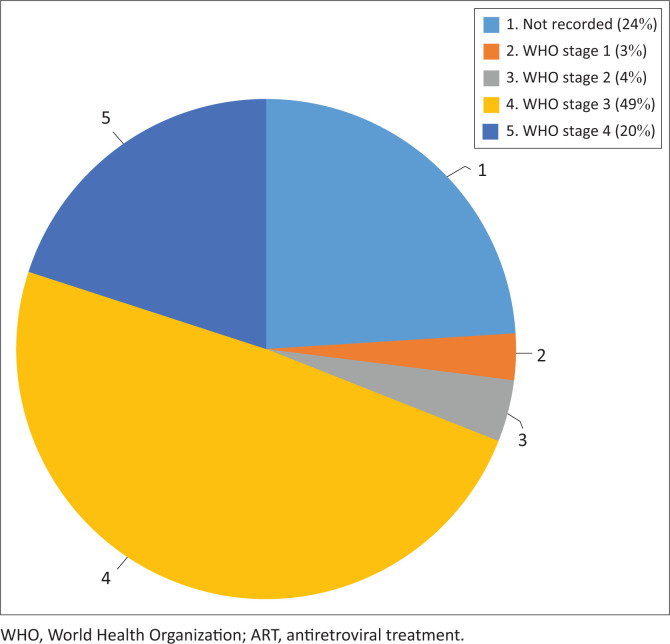
Staging of the infants and children included in the study according to World Health Organization clinical staging of HIV/AIDS for infants and children with confirmed HIV infection.

**FIGURE 3:**
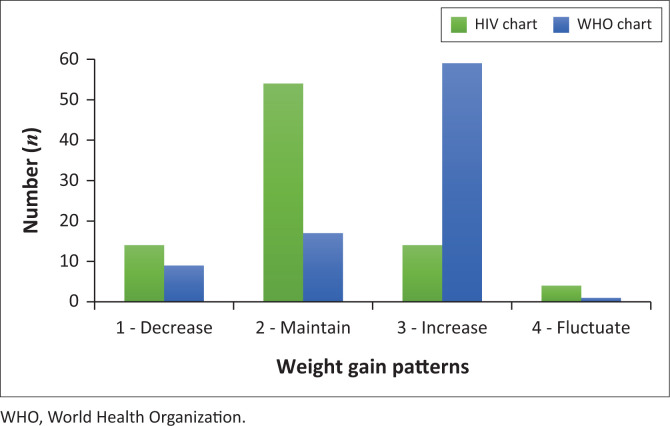
Weight gain patterns according to two references: interpretation according to HIV-specific weight gain curves versus weight-for-age z-scores (WHO charts).

**FIGURE 4:**
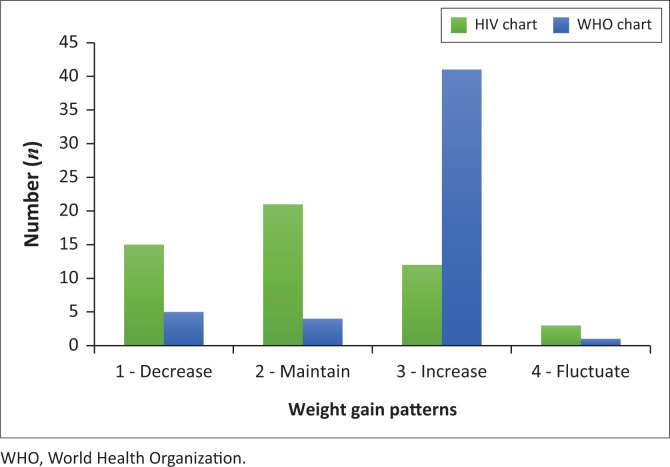
Subgroup analysis of participants with low baseline weight at ART initiation: Weight gain patterns according to two references: interpretation according to HIV-specific weight gain curves versus weight-for-age z-scores (WHO charts).

Figure 4 depicts a separate subgroup analysis that included only the participants who had a low WAZ at initiation of ART (*n* = 51, WAZ < −1). There was an improvement in the agreement between weight gain patterns between the two weight gain charts (HIV-specific weight gain curves versus the WHO growth charts), but the agreement was still not statistically significant (kappa value = 0.03; *P* = 0.58).

## Discussion

We found that more than half of the children in this study were already stunted and underweight for their age when they started ART, according to the WHO growth standards and references.^22,23^ These findings are in line with those of the literature, describing the association between growth and HIV infection and the tendency of CLHIV to be stunted by as early as three months of age.^28^ In this study, the children were on average already almost three years old when ART was initiated. Most of the study participants were clinically at Stage 3 and Stage 4 of HIV infection (69%), indicating advanced HIV disease progression.^27^ More recently progress has been made in ensuring that children are initiated on ART as soon as possible, due to vigorous testing for HIV during pregnancy.^29,30^

Research suggests that children with more advanced disease progression and associated poor growth at ART initiation may show larger improvements in weight gain when compared to children who weigh more at initiation of ART.^31,32,33^ This may have contributed to a threshold effect and also served as the justification behind the subgroup analysis that was performed in this study. The aim was to assess whether the participants who were at a low baseline weight (WAZ < −1) showed different growth patterns from the total group. Results indicated that the growth pattern of the subgroup (*n* = 51) versus the growth pattern of the entire group (*n* = 98) was similar, although most participants showed an increase in rate of weight gain. This observation may be explained by the high proportion (53.1%) of the participants who were underweight at baseline (WAZ < −2).

Improvements in haemoglobin levels, as well as in CD4% were significant over the 24-month period following ART initiation, indicating the positive effect of ART on general health and immunity. These results are in agreement with the findings of other studies in Africa.^34,35^

Statistically significant improvements in weight gain over the 24-month research period and at each six-month follow-up visit since ART was initiated was observed. Although the focus was on changes in WAZ, there was consistent improvement in both WAZ and L/HAZ, with more rapid increases in weight as opposed to gradual increases in length or height. This suggests that weight gain improves before height gain. Weight gain improved significantly from as early as six months following ART initiation, whereas significant improvements in linear growth were observed after 12 or more months of treatment. Similar growth reconstitution findings were observed in CLHIV on ART in other sub-Saharan African countries.^36^ The children in this study did not reach complete catch-up growth after 24 months of ART initiation, as the mean WAZ was still not optimal (WAZ = −1.1). The proposal that catch-up growth is cohort-specific was suggested, because generally cohorts of children in socio-economically advantaged regions have reached complete catch-up growth after two years on ART.^37^

This study was conducted in a resource-limited setting, where routine monitoring of viral load was not done at the time, although such measurements were done in better-resourced hospitals. Growth parameters and time to catch-up growth differ in resource-limited settings, where a high prevalence of malnutrition exists.^38^ According to the WHO Drug Resistance report 2012, Zambia was the only country reporting general viral load survey data from children and adolescents receiving ART at the time.^39^

The use of ART has a positive impact on the nutritional status of CLHIV, although studies have revealed a higher prevalence of wasting, underweight and stunting in this population, when compared to country statistics.^38^ It is not uncommon that only children with established malnutrition, as evidenced by anthropometrical measurements (weight, height and MUAC), are referred to dieticians for nutritional support and education, while children with ‘normal’ anthropometry are usually not referred.^40^

This study aimed to compare the weight gain patterns of the same group of infants and children according to two weight assessment charts: HIV-specific weight gain curves, proposed by Yotebieng et al.,^20^ and the WHO WAZ standard and reference charts.^22,23^ Results indicated that there is poor agreement between the assessment findings based on these two reference charts. The WHO growth standard and reference charts indicated that the largest proportion of the infants and children in this study had an increase in their rate of weight gain, whereas the HIV-specific weight gain curves showed that the largest proportion actually only maintained their rate of weight gain. Of special interest was the differences in the number of children that showed improvements in weight gain after ART initiation: 69% according to the WHO standard and reference charts, but only 16% according to the HIV-specific curves. This lack of agreement suggests that children’s weight gain may be over-estimated when applying the WHO growth charts in the clinical setting.

The results support the research conducted by Yotebieng et al.^20^ who suggest the use of population-specific weight gain charts for CLHIV and on ART, not only for correct referral and support purposes, but also to help understand the effects of different ART regimens on weight gain. The WHO growth standard charts were constructed using growth reference data of exclusively breastfed infants and children from six socio-economically advantaged regions.^22^ One should consider the significant variability in child growth across different populations, such as in CLHIV compared to healthy children. It is unlikely that any single framework can serve as a universal model for healthy growth. In addition, perceptions of caregivers about normal, healthy growth have significant implications for medical treatment and feeding practices.^41^ The need for disease- and treatment-specific growth curves should be investigated for the assessment of growth in children on ART.^20^ Better awareness of true growth issues in CLHIV initiated on ART can potentially help to avoid the possibility of CLHIV developing more severe malnutrition. Improved nutritional care has the capacity to influence disease outcomes, particularly among the vulnerable malnourished population, while malnutrition has long-lasting effects with potential impacts on the health of future generations.^42^

A limitation of this study was the retrospective design and therefore the reliance on measurements and record-keeping that could not be controlled and which could be susceptible to technical and human error. The impact of different ART regimens on the rate of weight gain could not be assessed due to the small sample size. The data were collected in a different era of ART and much has been done to improve initiation age and clinical management of CLHIV; however, there exists an increasing need for dietary counselling and the provision of nutritional education or supplementation at ART initiation in order to improve growth and development as well as health outcomes.^43^

More research is needed to determine the impact of nutrition interventions, especially at early stages of improper weight gain, in order to assess the effect on ART success. Improved monitoring of growth in CLHIV according to a HIV-specific reference could help to optimise the response to ART.^18^

## Conclusion

In this study, the weight gain improvements were observed among infants and children who were initiated on ART before the age of 10 years old. However, the children failed to reach complete catch-up growth after the 24-month study period. In addition, the weight gain patterns of the children varied greatly between the two growth charts that we compared. Weight gain of CLHIV on ART should be monitored according to curves that represent the population, because anthropometrical interpretations are widely used as a scale to evaluate individual and population growth status^25^ and their interpretations have important implications for child health programmes.^44^

Future research should aim to investigate the feasibility of applying population- or disease-specific growth charts for a larger sample of CLHIV who get exposed to ART to monitor the impact of ART exposure on their growth. Strategies to improve the nutritional practices in CLHIV may impact long-term growth outcomes in this vulnerable population.^45^
